# Nivolumab monotherapy or combination therapy with ipilimumab for lung cancer: a systemic review and meta-analysis

**DOI:** 10.1186/s12935-021-02100-w

**Published:** 2021-08-14

**Authors:** Huihui Jiang, Aiqun Xu, Wanli Xia, Xingyuan Xia, Pulin Li, Binbin Zhang, Ke Zhu, Sijing Zhou, Ran Wang

**Affiliations:** 1 Department of Respiratory and Critical Care Medicine, The First Affiliated Hospital of Anhui Medical University, Hefei, 230022 China; 2 Department of General Medicine, Hefei Second People’s Hospital, Hefei, 230001 China; 3 Department of Thoracic Surgery, The First Affiliated Hospital of Anhui Medical University, Hefei, 230022 China; 4 Hefei Third Clinical College of Anhui Medical University, Hefei, 230022 China; 5 Hefei Prevention and Treatment Center for Occupational Diseases, Hefei, 230022 China

**Keywords:** Antitumor drugs, Ipilimumab, Lung cancer, Nivolumab, Immunotherapy

## Abstract

**Background:**

The high incidence and mortality of lung cancer have seriously affected human life and health. Nivolumab is a monoclonal antibody that can inhibit programmed death 1 (PD-1) and Ipilimumab is a monoclonal antibody against CTLA-4(cytotoxic T lymphocyte-associated antigen 4), both of which can prevent the immune escape of tumor cells. Our goal was to synthesize evidence from published randomized controlled trials involving the safety and efficacy of either Nivolumab alone or in combination for the treatment of unresectable lung cancer.

**Methods:**

We searched the following electronic databases: PubMed, Embase, and Cochrane libraries, and screened the retrieved records for eligibility. We used the Stata16 software for the analyses. The results of the analysis are expressed as hazard ratios (HRs) or risk ratios (RRs) and their corresponding 95% confidence intervals (CI).

**Results:**

The final analysis included seven trials involving 3817 patients. Among patients with advanced lung cancer, patients using immunotherapy had better overall survival (OS), progression-free survival (PFS), and an objective response rate (ORR) than patients receiving chemotherapy. The HR of Nivolumab monotherapy or combination therapy with OS was compared with that of chemotherapy (HR: 0.73, 95% CI 0.64–0.83; HR: 0.67, 95% CI 0.55–0.81), and the HR of PFS was (HR: 0.81, 95% CI 0.69–0.94; HR: 0.67, 95% CI 0.55–0.82).

**Conclusions:**

Immunotherapy has been shown to have more clinically meaningful survival benefits for patients with lung cancer, whether monotherapy or combination immunotherapy.

CRD42020213440

## Background

Lung cancer is the most common malignant tumor and the most common cause of cancer-related death worldwide [1]. In 2017, there were 2.2 million cases of trachea, bronchus, and lung cancer in the world, with 1.9 million deaths, accounting for approximately 19% of all cancer-related deaths [2]. The high mortality [3] rate and low 5-year survival rate [4] of lung cancer have led to effective cancer treatments, which have directed lung cancer research. Systemic cytotoxic chemotherapy has been the main treatment for advanced non-small cell lung cancer (NSCLC), but the efficacy of chemotherapy is limited and new forms of treatment are required. Although there have been increasingly more research on alternative treatments for gene mutations, their success is still limited [5, 6]. Thus, we have again turned our attention to immunotherapy. Tumors can escape immune surveillance in the body because of changes within themselves or the tumor microenvironment, or through immune regulatory mechanisms, that is, the internal regulatory mechanism by which tumors induce or suppress immune responses [7]. Immunotherapy mainly regulates the interaction between T cells and antigen-presenting cells (APC) or tumor cells to help release suppressed immune responses [8]. At present, immunotherapy has become an important treatment method [9].

At present, the immune checkpoint inhibitors approved by the FDA for the treatment of non-small cell lung cancer are PD1/PD-L1 inhibitors and CTLA-4 inhibitors. Immune-monotherapy had a manageable safety profile, but the efficacy is limited by low response rates [10, 11]. While the results of monotherapy treatments are not satisfactory, there is increasing emphasis on combination treatments in an effort to increase response rates to treatment. Nivolumab, a fully human anti–PD-1 antibody, and ipilimumab, a fully human anti–cytotoxic T-lymphocyte antigen 4 (CTLA-4) antibody, are immune checkpoint inhibitors with distinct but complementary mechanisms of action [12]. Combination therapy with nivolumab plus ipilimumab has resulted in longer overall survival than previous standard therapies in patients with melanoma [13] and in those with renal-cell carcinoma [14]. But in lung cancer, the combination of ipilimumab and nivolumab has not been approved by FDA.

Nivolumab (BMS-936558) was the first PD-1 inhibitor approved for advanced NSCLC [15]. Previous studies have found that the pharmacokinetics of Nivolumab is linear in the dose range of 0.1 to 20 mg/kg. Additionally, after drug withdrawal, receptor saturation can be maintained for several months [16, 17]. In a multicenter phase one dose-escalation cohort expansion trial [18], 129 patients with advanced NSCLC were evaluated and Nivolumab was divided into three dose groups: 1 mg/kg, 3 mg/kg, and 10 mg/kg. The 5-year results showed that the objective response rate (ORR) and median overall survival (OS) of patients treated with Nivolumab was 17.1% and 9.9 months, respectively. The 3 mg/kg dose group had the best effect wherein the median OS reached 14.9 months. This 5-year follow-up study showed that the 5-year OS rate for all patients was 16%, and the 5-year OS rate for squamous cell carcinoma (16%) and non-squamous cell carcinoma (15%) was similar. In 2015, Nivolumab was approved (FDA) for the treatment of advanced metastatic squamous and non-squamous NSCLC after platinum chemotherapy [18].

Ipilimumab was approved by the FDA in 2011 for the treatment of unresectable or metastatic melanoma [19]. In a multicenter phase II study of small-cell lung cancer, the effects on patients were compared for those receiving chemotherapy alone or in combination with Ipilimumab. Compared with the control group, the Ipilimumab group had obvious advantages in immune-related progression-free survival (irPFS) and OS [19]. Larkin found that for patients with previously untreated metastatic melanoma, the survival rate of patients receiving Nivolumab plus Ipilimumab was significantly improved when compared with those receiving Ipilimumab alone [20].

In this meta-analysis, our goal was to synthesize the evidence of published randomized controlled trials (RCTs) to study the safety and effectiveness of Nivolumab monotherapy and that of Nivolumab combined with Ipilimumab.

## Materials and methods

This meta-analysis was based on PRISMA guidelines. The studies included in this meta-analysis have been published, and, as such, ethical approval and informed consent were not required.

### Inclusion and exclusion criteria

We included RCTs that met the following criteria: (i) population: patients with advanced stage III or IV lung cancer; (ii) intervention: Nivolumab alone or in combination with Ipilimumab; (iii) control: Nivolumab monoclonal antibody or any other effective chemotherapy; (iv) results: objective response rate, complete response rate, partial response rate, PFS rate, and safety results. We excluded tests that met the following conditions: (i) non-English publications and (ii) conference papers. In multiple reports, we used the data from the latest report.

### Search strategy

We searched electronic databases for documents published as of December 2020. The databases included Embase, Cochrane Library, and PubMed. We used an advanced search for all databases. Search criteria used (Opdivo [title/abstract]), (Nivolumab [title/abstract]), (Ipilimumab [title/abstract]), and (non-small cell lung cancer [Title/Abstract]), (NSCLC [Title/Abstract]), (lung cancer [Title/Abstract]), or (small cell lung cancer [title/abstract]).

### Data extraction

**Fig. 1:**
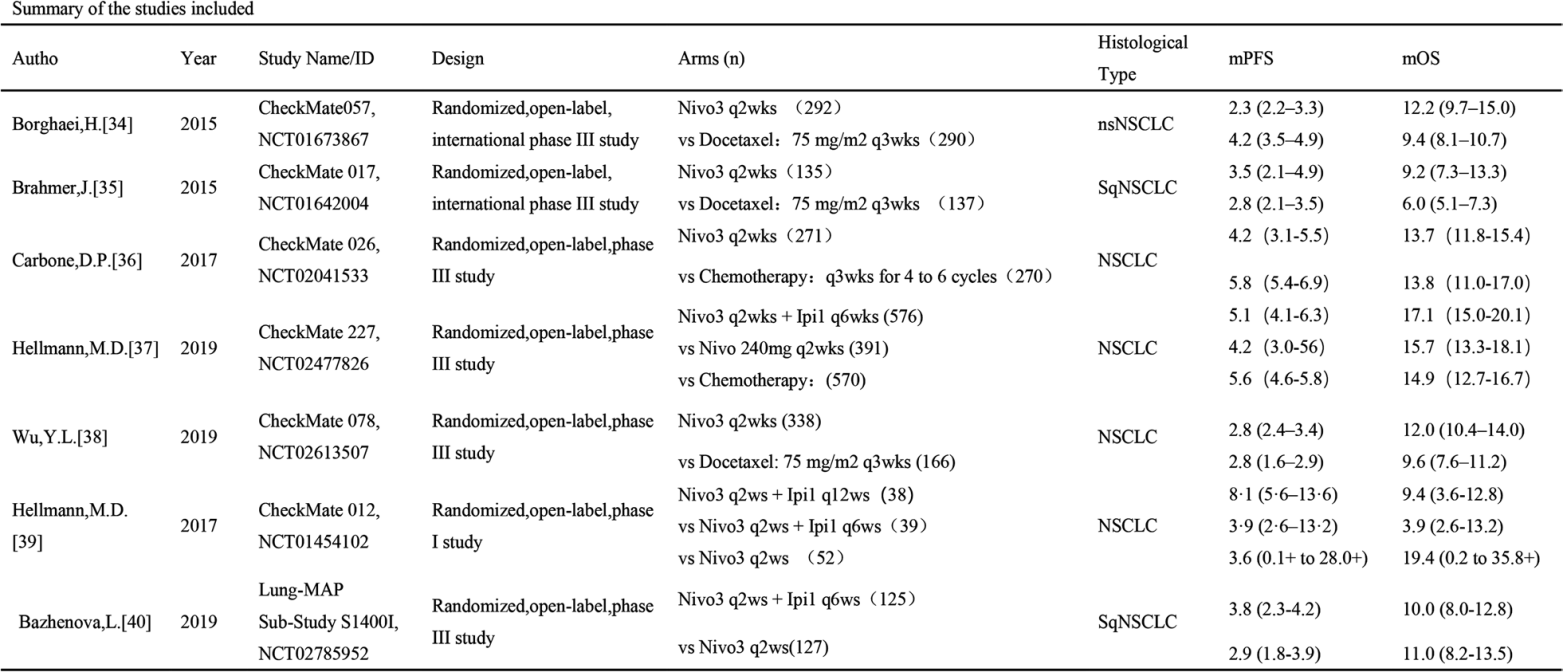
Studies included in this article

Two reviewers extracted data from qualified studies and differences were resolved with a discussion. The name of the first author and the year of publication were used to identify the study. For each study, the following information was extracted: first author name, annual publications, experimental stage, number of subjects, interventions, prognosis (OS, PFS, adverse effects [AEs], ORR). The characteristics of the included studies are shown in Fig. 1.

### Quality assessment

The Cochrane Collaboration tool was used to assess the risk of RCT bias, considering the following aspects: whether the method of allocation was truly random, whether proper concealment of allocation existed, whether equality occurred between the two groups at baseline in terms of prognostic features, whether the eligibility criteria were described, whether blinding of the outcome assessors was performed, whether the report was complete, and whether the report was selective (Table 1). A funnel plot and Harbord’s test were used to assess publication bias.

**Table 1:** Quality assessment by the cochrane collaboration’s tool

Reference	Random Sequence generation	Allocation concealment	Blinding of participants and personnel	Blinding of outcome assessment	Incomplete outcome data	Selective reporting	Other bias
Borghaei, H. [34]	Unclear risk	Unclear risk	Unclear risk	Low risk	Low risk	Unclear risk	Unclear risk
Brahmer, J. [35]	Unclear risk	Unclear risk	Unclear risk	Unclear risk	Low risk	Unclear risk	Unclear risk
Carbone, D. P. [36]	Unclear risk	Unclear risk	Unclear risk	Low risk	Low risk	Unclear risk	Unclear risk
Hellmann, M. D. [33]	Unclear risk	Unclear risk	Unclear risk	Low risk	Unclear risk	Unclear risk	Unclear risk
Wu, Y. L. [38]	Low risk	Unclear risk	Unclear risk	Unclear risk	Unclear risk	Unclear risk	Unclear risk
Hellmann, M. D [39]	Low risk	Unclear risk	Unclear risk	Unclear risk	Unclear risk	Unclear risk	Unclear risk
Bazhenova, L [40]	Unclear risk	Unclear risk	Unclear risk	Unclear risk	Unclear risk	Unclear risk	Unclear risk

### Statistical analysis

All analyses were performed using Stata 16.0 and ICT (Indirect Treatment Comparisons, Canadian Agency for Drugs and Technologies in Health). The results of the meta-analysis were expressed as hazard ratios (HR) or risk ratios (RRs) and their corresponding 95% CI. PFS and OS were expressed with HRs and corresponding 95% CI. The RR and corresponding 95% CI were the comprehensive measure of the risk of ORR and AEs. We first tested heterogeneity among studies using I^2^ statistics and I^2^ < 50% was considered a low level of heterogeneity, whereas I^2^ ≥ 50% indicated a high level of heterogeneity. Based on the statistical significance of the heterogeneity test, we used a random effects model (I^2^ ≥ 50%) or a fixed effects model (I^2^ < 50%) to calculate the combined results. Metaninf and meta-regression were used to explore the sources of heterogeneity.

## Results

**Fig. 2:**
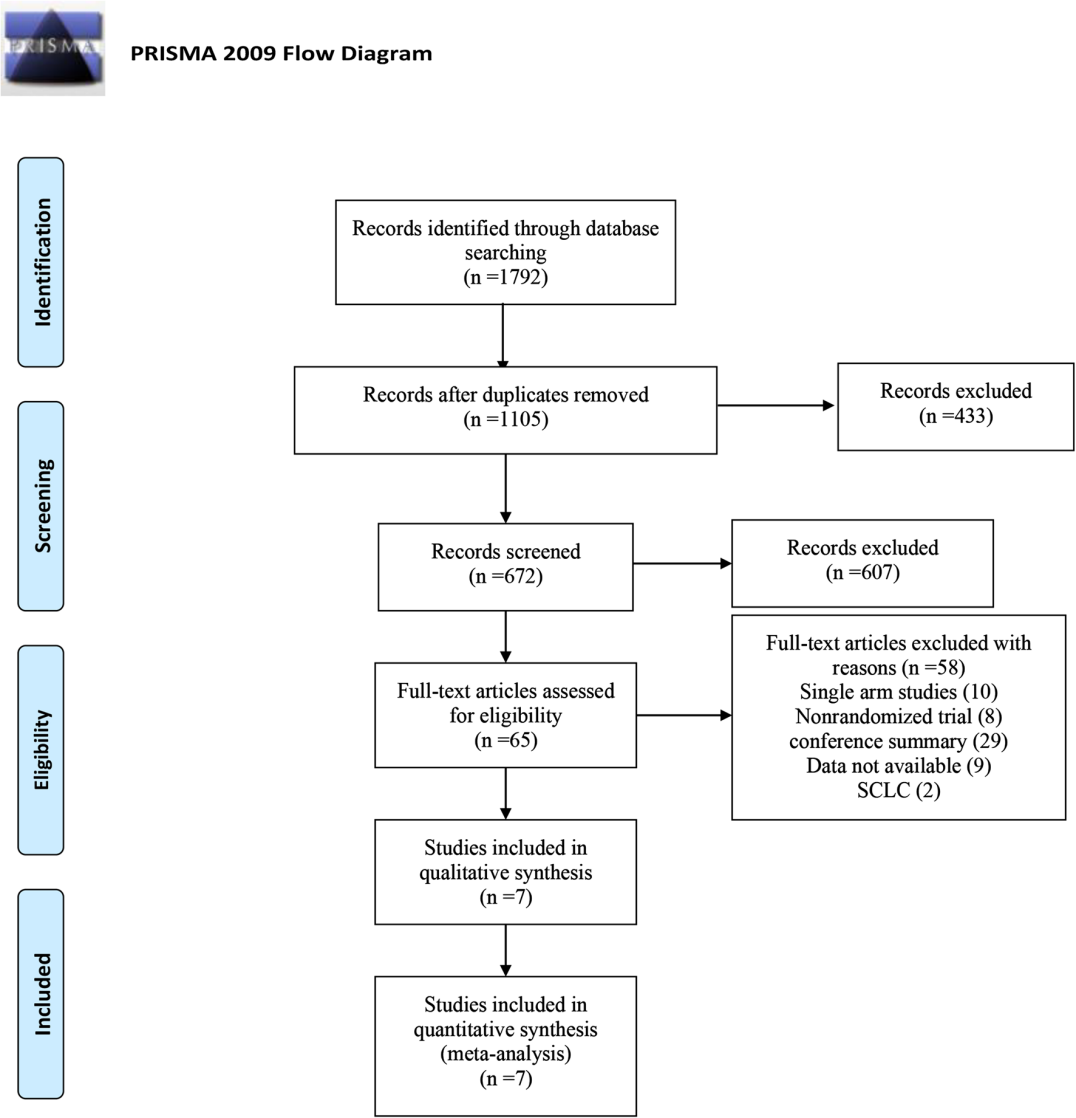
Flow diagram of the literature search and trial selection process

**Fig. 3:**
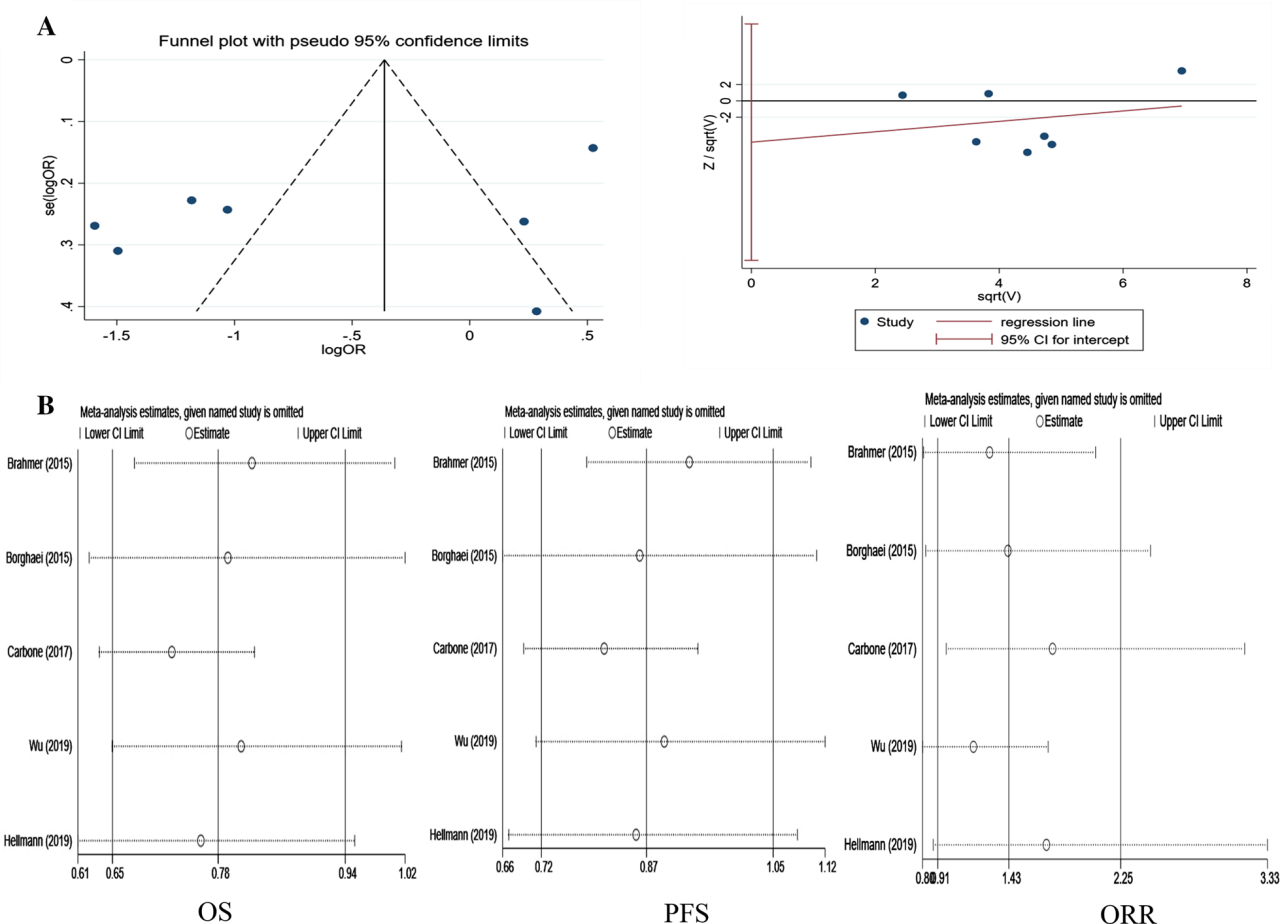
Publication bias and sensitivity analysis. **A** Detection of publication bias; **a** funnel plot; **b** Egger’s test; **B** sensitivity analysis of overall survival, progression-free survival, and objective response rate. **a** Nivolumab monotherapy versus chemotherapy in OS; **b** Nivolumab monotherapy versus chemotherapy in PFS; **c** Nivolumab monotherapy versus chemotherapy in ORR

The initial search resulted in 1792 records from the Cochrane Library, Embase, and PubMed. Among them, 687 studies were deleted as duplicate records, and 65 potential studies were identified as full-text reviews. Among them, the single-arm test was excluded because of the lack of a control group. Seven RCTs involving 3817 patients met the inclusion criteria and were included in this meta-analysis. Figure 2 details the selection process. Publication bias detection was performed for OS analysis. The funnel plot showed there may be incomplete symmetry; therefore, we conducted the Harbord’s test (Fig. 3A). The Harbord’s test (|P|= 0.410 > 0.05) showed no publication bias.

### OS of nivolumab plus ipilimumab or nivolumab alone

**Fig. 4:**
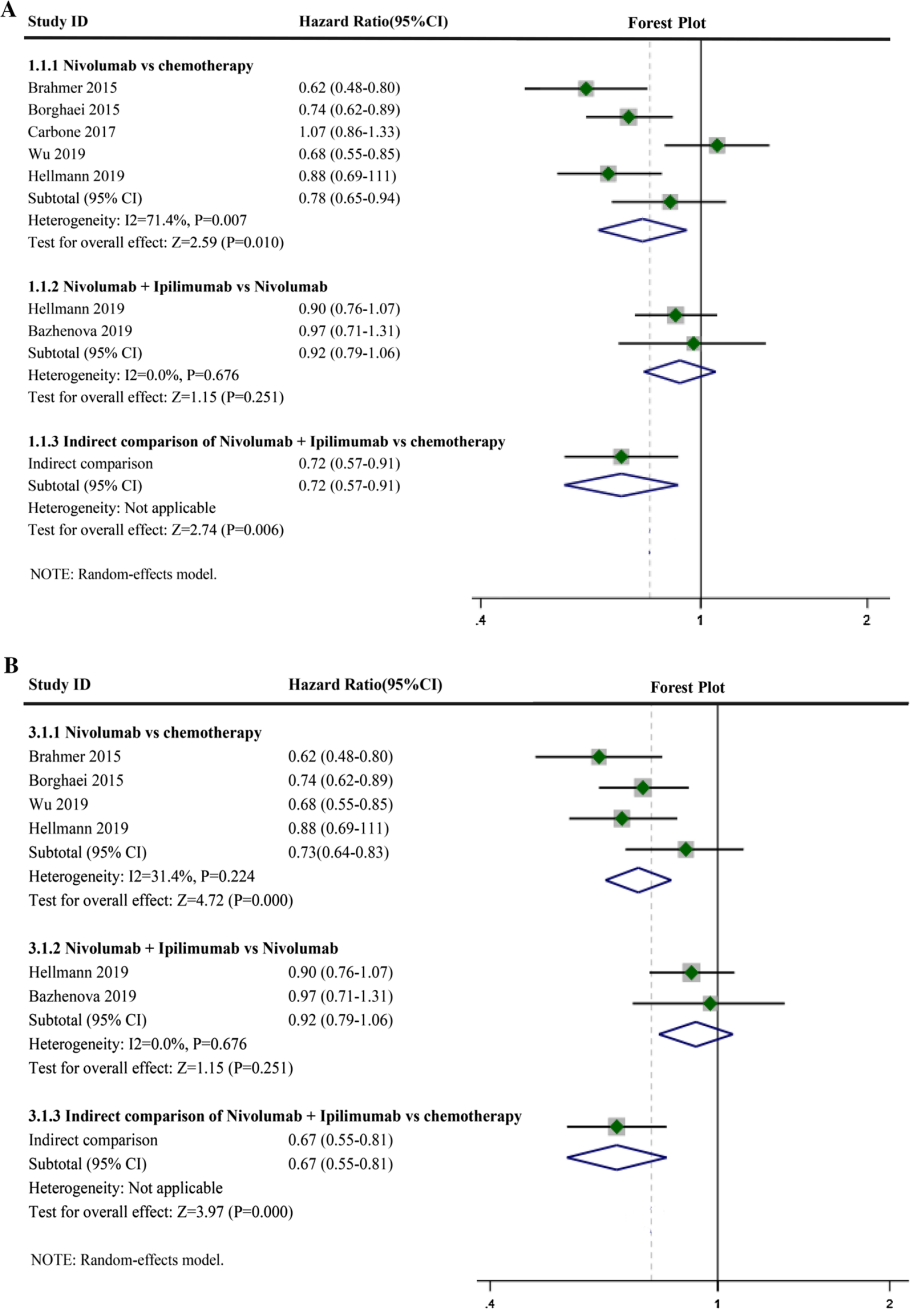
Forest plots of overall survival (OS). **A** Forest plots of overall survival (OS) for Nivolumab alone or in combination with Ipilimumab; CI, confidence interval; **B** Forest plots of OS for Nivolumab alone or in combination with Ipilimumab after removing one study; CI, confidence interval

Compared with chemotherapy, Nivolumab monotherapy benefited OS (Fig. 4A, HR: 0.78, 95% CI 0.65–0.94, I^2^ = 71.4%, P = 0.007), and the combination of Nivolumab and Ipilimumab also had a beneficial effect compared with that of Nivolumab monotherapy (Fig. 4A, HR: 0.92, 95% CI 0.79–1.06, I^2^ = 0.0%, P = 0.676). A direct comparison using ICT showed the OS of Nivolumab and Ipilimumab combination therapy compared with that of chemotherapy (HR: 0.72, 95% CI: 0.57–0.91). Heterogeneity of the Nivolumab monotherapy group was I^2^ > 50%. Sensitivity analysis was performed, which suggested that Carbone's (2019) study may be the source of heterogeneity (Fig. 3B-OS). Acceptable heterogeneity was obtained when Carbone’s (2019) study was excluded (Fig. 4B, HR: 0.73, 95% CI 0.64–0.83, I^2^ = 31.4%, P = 0.224). A direct comparison using ICT showed the OS of Nivolumab and Ipilimumab combination therapy compared with that of chemotherapy (HR: 0.67, 95% CI 0.55–0.81).

### PFS of nivolumab plus ipilimumab and nivolumab alone

**Fig. 5:**
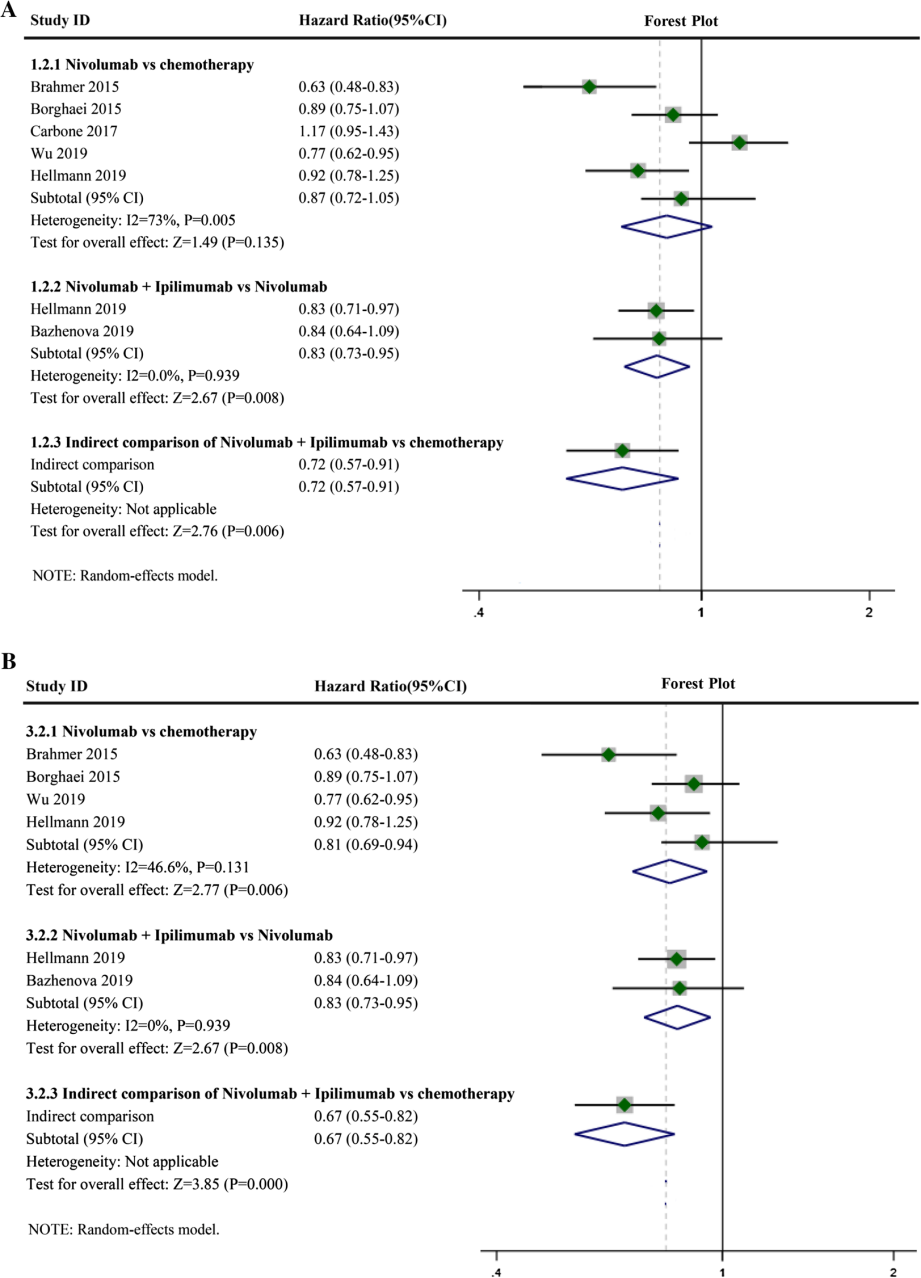
Forest plots of progressive-free survival (PFS). **A** Forest plots of PFS for Nivolumab alone or in combination with Ipilimumab; CI, confidence interval; **B** Forest plots of PFS for Nivolumab alone or in combination with Ipilimumab after removing one study; CI, confidence interval

Compared with chemotherapy, Nivolumab monotherapy benefited PFS (Fig. 5A, HR: 0.87, 95% CI 0.72–1.05, I^2^ = 73.0%, P = 0.005), and the combination of Nivolumab and Ipilimumab also was beneficial compared with that of Nivolumab monotherapy (Fig. 5A, HR: 0.83, 95% CI 0.73–0.95, I^2^ = 0.0%, P = 0.939). A direct comparison using ICT showed the PFS of Nivolumab and Ipilimumab combination therapy compared with that of chemotherapy (HR: 0.72, 95% CI 0.57–0.91). The heterogeneity of the Nivolumab monotherapy groups was I^2^ > 50%. Sensitivity analysis was performed, suggesting that Carbone’s (2019) study may be the source of heterogeneity (Fig. 3B-PFS). Acceptable heterogeneity was obtained when Carbone's (2019) study was excluded (Fig. 5B, HR: 0.81, 95% CI 0.69–0.94, I^2^ = 46.6%, P = 0.131). A direct comparison using ICT showed the PFS of Nivolumab and Ipilimumab combination therapy compared with that of chemotherapy (HR: 0.67, 95% CI 0.55–0.82).

### ORR of Nivolumab plus Ipilimumab or Nivolumab alone

**Fig. 6:**
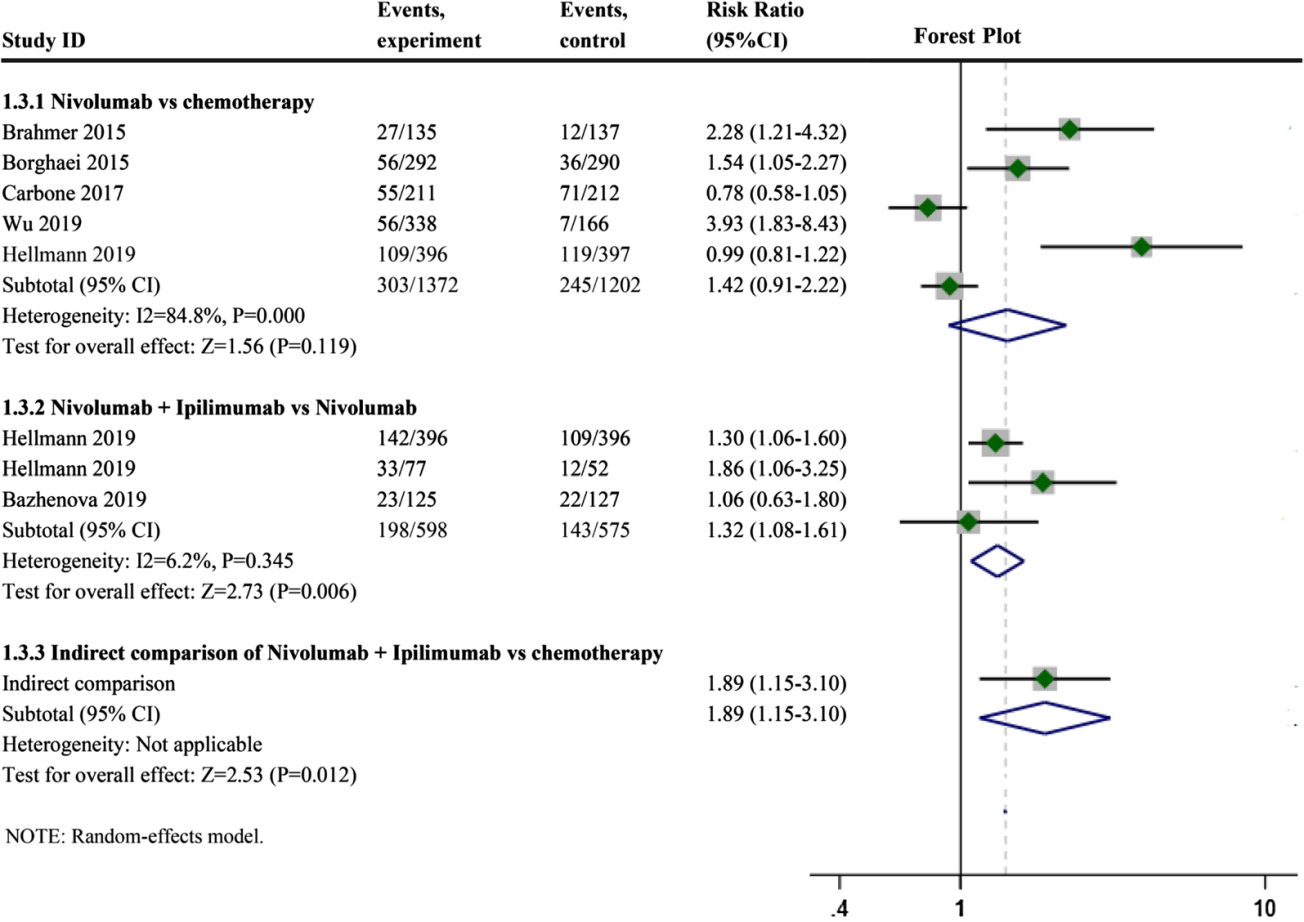
Forest plots of objective response rate (ORR). Forest plots of ORR for Nivolumab alone or in combination with Ipilimumab; CI, confidence interval

The Nivolumab monotherapy group had a higher ORR than the chemotherapy group (Fig. 6, RR: 1.42, 95% CI 0.91–2.22, I^2^ = 84.8%, P = 0.000), and the ORR of Nivolumab combined with Ipilimumab was higher than that of Nivolumab monotherapy (Fig. 6, RR: 1.32, 95% CI 1.08–1.61, I^2^ = 6.2%, P = 0.345. A direct comparison using ICT showed the ORR of Nivolumab and Ipilimumab combination therapy compared with that of chemotherapy (RR: 1.89, 95% CI: 1.15–3.10). The heterogeneity of the Nivolumab monotherapy groups was I^2^ > 50%. We failed to find the source of heterogeneity by sensitivity analysis (Fig. 3C-ORR). Meta-regression was conducted to detect the source of heterogeneity, which may be related to the PS score of patients in the study. Meta-regression results showed the more people with a high PS score in a given study, the better the ORR.

**Fig. 7:**
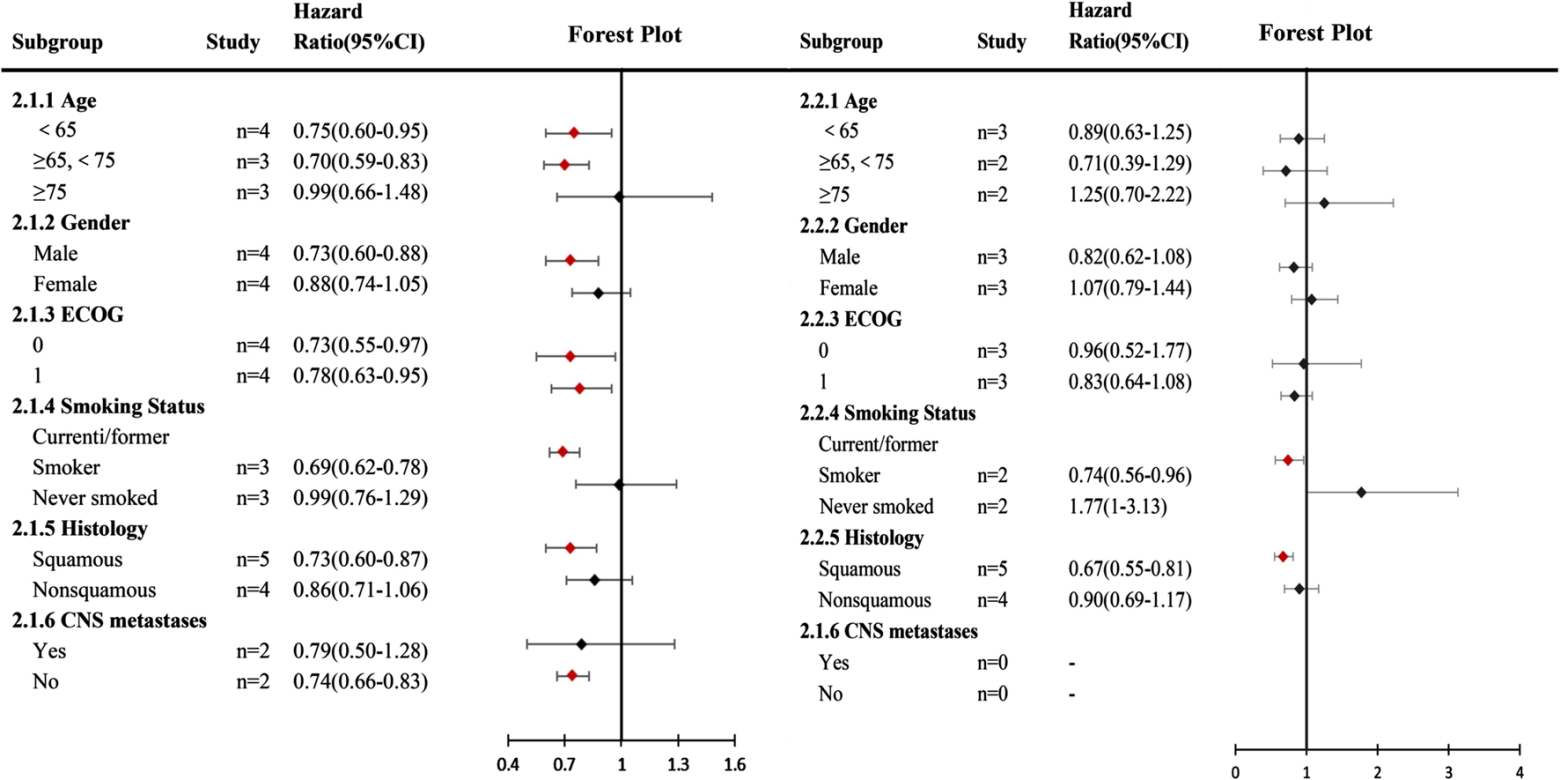
Subgroup meta-analysis of progression-free survival (PFS) and overall survival (OS). (2.1) Nivolumab alone or in combination with Ipilimumab versus chemotherapy in PFS; (2.2) Nivolumab alone or in combination with Ipilimumab versus chemotherapy in OS. CI: 95% confidence interval

Compared with the chemotherapy group, patients with lung squamous cell carcinoma and current/former smokers had better OS (HR: 0.73,0.60–0.87; HR: 0.69,0.62–0.78) and PFS (HR: 0.67,0.55–0.81; HR: 0.74,0.56–0.96) in the nivolumab monotherapy group (Fig. 7). Similarly, the use of nivolumab monotherapy can also achieve better OS in patients with age < 75 (HR: 0.75,0.60–0.95; HR: 0.70,0.59–0.83), male (HR: 0.73,0.60–0.88), ECOG <  = 1 (HR: 0.73,0.55–0.97; HR: 0.78,0.63–0.95) and no CNS metastasis (HR: 0.74,0.66–0.83) (Fig. 7).

Compared with chemotherapy, Nivolumab exhibited statistical significance in all grades of AEs, high-grade AEs (Table 2), and compared with Nivolumab, Nivolumab combined with Ipilimumab exhibited statistical significance in all grades of AEs and high-grade AEs (Table 3). The risk of high-grade and all-grade Fatigue was statistically higher for nivolumab vs chemotherapy (Table 2). The risk of all-grade Nausea, Vomiting, appetite, Constipation, Neutrophil count decreased, Neutropenia, Anemia, Leukopenia and WBC count decreased were statistically higher for nivolumab vs chemotherapy (Table 2). The risk of high-grade and all-grade Discontinuation was statistically lower for combined therapy vs. nivolumab (Table 3). The risk of all-grade Pruritis, Hypothyroidism, Nausea, Decreased appetite, Constipation and Diarrhea were statistically lower for combined therapy vs. nivolumab (Table 3).

**Table 2:** Pooled relative risk of all grade and high grade irAEs for nivolumab vs chemotherapy

All grade irAEs	No. of trials	Pooled RR (95% CI)	Pooled RR	p Value	High grade irAEs	No. of trials	Pooled RR (95% CI)	Pooled RR	p Value
Total incidence	4	0.728–0.805	0.765	0.000	Total incidence	4	0.149–0.324	0.220	0.000
Discontinuation	1	0.454–1.180	0.732	0.200	Discontinuation	1	0.657–2.254	1.217	0.533
Fatigue	4	0.440–0.627	0.525	0.000	Fatigue	4	0.103–0.418	0.208	0.000
Rash	3	0.647–4.516	1.71	0.279	Rash	3	0.248–5.904	1.210	0.814
Nausea	3	0.221–0.534	0.344	0.000	Nausea	3	0.107–1.447	0.394	0.160
Vomiting	1	0.144–0.422	0.246	0.000	Vomiting	1	0.005–1.611	0.090	0.102
Decreased appetite	4	0.345–0.643	0.471	0.000	Decreased appetite	4	0.071–1.387	0.315	0.127
Constipation	1	0.148–0.633	0.306	0.001	Constipation	1	–	–	–
Diarrhea	3	0.231–1.172	0.52	0.115	Diarrhea	3	0.178–1.424	0.504	0.196
Pneumonia	1	0.729–224.957	12.803	0.081	Pneumonia	1	–	–	–
Arthralgia	1	0.294–1.995	0.766	0.585	Arthralgia	1	–	–	–
Neutrophil count decreased	2	0.018–0.111	0.045	0.000	Neutrophil count decreased	2	0.004–0.149	0.025	0.000
Neutropenia	4	0.005–0.144	0.028	0.000	Neutropenia	4	0.005–0.066	0.019	0.000
Anemia	4	0.075–0.161	0.11	0.000	Anemia	4	0.024–0.228	0.075	0.000
Leukopenia	3	0.028–0.435	0.11	0.002	Leukopenia	3	0.012–0.225	0.053	0.000
WBC count decreased	1	0.059–0.225	0.116	0.000	WBC count decreased	1	0.113–0.253	0.169	0.000

**Table 3:** Pooled relative risk of all grade and high grade irAEs for combination vs nivolumab

All grade irAEs	No. of trials	Pooled RR (95% CI)	Pooled RR	p Value	High grade irAEs	No. of trials	Pooled RR (95% CI)	Pooled RR	p Value
Total incidence	2	1.067–1.254	1.157	0.000	Total incidence	2	1.337–2.089	1.672	0.000
Discontinuation	2	1.062–1.975	1.448	0.019	Discontinuation	1	1.133–2.656	1.735	0.011
Fatigue	2	0.550–1.660	0.956	0.872	Fatigue	2	0.720–11.245	2.845	0.136
Pruritis	2	1.320–2.736	1.900	0.001	Pruritis	2	0.245–91.337	4.731	0.304
Rash	2	0.508–2.391	1.102	0.806	Rash	2	0.491–4.239	1.442	0.506
Hyperthyroidism	2	0.947–13.480	3.573	0.060	Hyperthyroidism	1	0.188–111.215	4.577	0.350
Hypothyroidism	2	1.170–2.828	1.819	0.008	Hypothyroidism	1	0.124–14.921	1.358	0.803
Nausea	2	1.066–2.482	1.626	0.024	Nausea	2	0.323–12.855	2.037	0.449
Vomiting	2	0.877–3.123	1.655	0.120	Vomiting	2	0.232–10.670	1.573	0.643
Decreased appetite	1	1.282–3.064	1.982	0.002	Decreased appetite	1	0.246–91.812	4.156	0.302
Constipation	1	1.069–6.332	2.602	0.035	Constipation	1	–	–	–
Diarrhea	2	1.092–2.037	1.491	0.012	Diarrhea	2	0.514–5.392	1.664	0.396
Pneumomitis	1	0.353–5.161	1.351	0.660	Pneumomitis	1	0.217–18.948	2.026	0.536

## Discussion

This study reports the results of a meta-analysis. Compared with chemotherapy, Nivolumab monotherapy or combination therapy with Ipilimumab has better efficacy. Additionally, compared with Nivolumab monotherapy, combination therapy had better efficacy. Nivolumab resulted in a significantly longer OS and a higher response rate. Furthermore, the Nivolumab group showed acceptable safety compared with the Ipilimumab combination group or the chemotherapy group.

In recent years, research on immune checkpoint inhibitors of PD-1, PD-L1, and CTLA-4 have become a hot topic in the field of cancer because of their remarkable efficacy in prolongation of the survival of patients with non-small cell lung cancer, melanoma, and renal cell carcinoma [21, 22]. In the PD-1/PD-L1 pathway, PD-1 can be expressed on T cells, B lymphocytes, and natural killer (NK) cells, among others [9, 23]. They combine with PD-L1 and PD-L2, resulting in T cell response in the tumor microenvironment [24]. A variety of tumor cells can express PD-L1, and PD-1 binds to PD-L1 to inhibit CD8 cytotoxic immune response and anti-tumor immune response, resulting in tumor immune tolerance [25]. Previous studies have found that PD-1 inhibitors are meaningful and safe for the survival of lung cancer patients [26]. The expression of PD-L1 is a predictive biomarker of ORR for advanced NSCLC using PD-1/PD-L1 inhibitors [27]. The CD28/CTLA-4 immunomodulatory system exists when CTLA-4 combines with B7 molecule antigen-presenting cells (APC), which can reduce T cell activity and prevent T cell activation channels from exerting immunosuppressive effects in tumors [28].

Nivolumab and Ipilimumab were the first combination of PD-1/CTLA-4, which has shown safety and superior efficacy in metastatic melanoma [20]. Long-term follow-up results from CheckMate 067 [29] showed that Nivolumab monotherapy or combination therapy with Ipilimumab improved patients' ORR, PFS, and OS. The 3-year and 4-year survival rates in the Nivolumab + Ipilimumab group were 58% and 53%, respectively. In Amr Menshawy’s study [30], Nivolumab alone or in combination with Ipilimumab was more effective than Ipilimumab alone or chemotherapy in melanoma patients. Nivolumab resulted in a significantly longer PFS and a higher response rate. The Nivolumab group showed acceptable safety compared with the Ipilimumab and chemotherapy groups. In 2016, Nivolumab and Ipilimumab were approved in the United States and the European Union for the treatment of non-primary melanoma. Clinical trials of combined immunotherapy in melanoma, lung cancer, and kidney cancer are still ongoing.

The MYSTIC [31] trial is a phase III trial of patients with lung cancer that uses the same strategy but different drugs, such as Durvalumab (anti-PD-L1) and Tremelimumab (anti-CTLA-4) as first-line therapy. This experiment included 1118 patients, and the median OS was 11.9 months (95% CI 9.0–17.7) with Durvalumab plus Tremelimumab (HR vs. chemotherapy, 0.85; 98.77% CI 0.61–1.17; P = 0.20). Median PFS was 3.9 months (95% CI 2.8–5.0) with Durvalumab plus Tremelimumab vs 5.4 months (95% CI 4.6–5.8) with chemotherapy (HR, 1.05; 99.5% CI 0.72–1.53; P = 0.71). ARCTIC [32]is another clinical trial evaluating the combination of Durvalumab and Tremelimumab compared to SOC chemotherapy. The results of the two studies suggested that Durvalumab alone or in combination with Tremelimumab had clinically significant improvement in OS and PFS compared with SOC. Safety was similar to that of previous studies.

Combination therapy seems to be the best strategy. However, we should also endeavor to find biomarkers, such as absolute lymphocyte count and tumor-infiltrating T cells to predict treatment response, which will not only contribute to the development of immunotherapy but may also achieve personalization of treatment.

CheckMate 568 (NCT02659059) [33] is a large single-arm phase 2 study of first-line Nivolumab plus Ipilimumab in the treatment of NSCLC. The primary endpoint was the objective mitigation rate (ORR) reviewed independently, and the secondary endpoint was the ORR analysis of TMB. The results showed that TMB ≥ 10 mut/Mb was related to the enhancement of the Nivolumab plus Ipilimumab treatment response regardless of the expression level of PD-L1, having an ORR > 40% [33]. Although TMB was measured in three experiments in this study, the defining point of TMB could not be unified; therefore, this study did not conduct a summary analysis of TMB.

Some of the results of our study were heterogeneous. In studies on OS and PFS, we found that the results were homogeneous after the elimination of the Carbone (2019) [36] study. Heterogeneity may be mainly derived from clinical heterogeneity. Compared with other studies (Brahmer (2015) [35], Borghaei (2015) [34], Wu (2019) [38], Hellmann (2019) [37]), docetaxel was not used in the control group in the Carbone (2019) study. The baseline characteristics of the patients were also different. In the Carbone (2019) study, 78.2% of people had PD1 expression ≥ 5%, which was higher than that in other studies (Brahmer, 2015; Borghaei, 2015; Wu, 2019; Hellmann, 2019). Additionally, fewer people in the study had an ECOG PS ≥ 1. Similar to other meta-analyses, our study had some limitations. The data were extracted from the summary data, not from individual patients in each trial. Therefore, the results of this analysis need to be treated with caution.

## Conclusion

Compared with chemotherapy, Nivolumab monotherapy and combination therapy can achieve longer OS, PFS and higher ORR, and the effect of combination therapy is more obvious. However, the risk of adverse reactions related to the combination therapy is higher than that of the Nivolumab monotherapy group. Nivolumab alone in patients with lung squamous cell carcinoma and smoking leads to longer OS, PFS and has acceptable safety.

## Data Availability

Not applicable.

## Abbreviations

PD-1: Programmed cell death-1
CTLA-4: Cytotoxic T lymphocyte-associated antigen 4
RR: Relative risk
OR: Odd ratio
HR: Hazard ratio
CI: Confidence intervals

## References

[1] Bray F, Ferlay J, Soerjomataram I (2018). Global cancer statistics 2018: GLOBOCAN estimates of incidence and mortality worldwide for 36 cancers in 185 countries. CA Cancer J Clin.

[2] Global Burden of Disease Cancer Collaboration (2019). Global, regional, and national cancer incidence, mortality, years of life lost, years lived with disability, and disability-adjusted life-years for 29 cancer groups, 1990 to 2017: a systematic analysis for the global burden of disease study. JAMA Oncol.

[3] Jemal A, Thun MJ, Ries LA (2008). Annual report to the nation on the status of cancer, 1975–2005, featuring trends in lung cancer, tobacco use, and tobacco control. J Natl Cancer Inst.

[4] Odahowski CL, Hebert JR, Eberth JM (2018). Regional variation in lung and bronchus cancer survival in the US using mortality-to-incidence ratios. Spat Spatiotemporal Epidemiol.

[5] Solomon BJ, Mok T, Kim DW (2014). First-line crizotinib versus chemotherapy in ALK-positive lung cancer. N Engl J Med.

[6] Lee CK, Brown C, Gralla RJ (2013). Impact of EGFR inhibitor in non-small cell lung cancer on progression-free and overall survival: a meta-analysis. J Natl Cancer Inst.

[7] Finn OJ (2012). Immuno-oncology: understanding the function and dysfunction of the immune system in cancer. Ann Oncol.

[8] Li X, Shao C, Shi Y (2018). Lessons learned from the blockade of immune checkpoints in cancer immunotherapy. J Hematol Oncol.

[9] Schalper KA, Brown J, Carvajal-Hausdorf D (2015). Objective measurement and clinical significance of TILs in non-small cell lung cancer. J Natl Cancer Inst.

[10] Rotte A, Jin JY, Lemaire V (2018). Mechanistic overview of immune checkpoints to support the rational design of their combinations in cancer immunotherapy. Ann Oncol.

[11] Rotte A (2019). Combination of CTLA-4 and PD-1 blockers for treatment of cancer. J Exp Clin Cancer Res.

[12] Hellmann MD, Paz-Ares L, Bernabe Caro R (2019). Nivolumab plus ipilimumab in advanced non-small-cell lung cancer. N Engl J Med.

[13] Larkin J, Chiarion-Sileni V, Gonzalez R (2019). Five-year survival with combined nivolumab and ipilimumab in advanced melanoma. N Engl J Med.

[14] Motzer RJ, Tannir NM, McDermott DF (2018). Nivolumab plus ipilimumab versus sunitinib in advanced renal-cell carcinoma. N Engl J Med.

[15] Wang L, Zhao D, Qin K (2019). Effect and biomarker of nivolumab for non-small-cell lung cancer. Biomed Pharmacother.

[16] Johnson DB, Peng C, Sosman JA (2015). Nivolumab in melanoma: latest evidence and clinical potential. Ther Adv Med Oncol.

[17] Brahmer JR, Drake CG, Wollner I (2010). Phase I study of single-agent anti-programmed death-1 (MDX-1106) in refractory solid tumors: safety, clinical activity, pharmacodynamics, and immunologic correlates. J Clin Oncol.

[18] Gettinger S, Horn L, Jackman D (2018). Five-year follow-up of nivolumab in previously treated advanced non-small-cell lung cancer: results from the CA209-003 study. J Clin Oncol.

[19] Reck M, Bondarenko I, Luft A (2013). Ipilimumab in combination with paclitaxel and carboplatin as first-line therapy in extensive-disease-small-cell lung cancer: results from a randomized, double-blind, multicenter phase 2 trial. Ann Oncol.

[20] Larkin J, Chiarion-Sileni V, Gonzalez R (2015). Combined nivolumab and ipilimumab or monotherapy in untreated melanoma. N Engl J Med.

[21] Postow MA, Chesney J, Pavlick AC (2015). Nivolumab and ipilimumab versus ipilimumab in untreated melanoma. N Engl J Med.

[22] Wolchok JD, Chiarion-Sileni V, Gonzalez R (2017). Overall survival with combined nivolumab and ipilimumab in advanced melanoma. N Engl J Med.

[23] Keir ME, Butte MJ, Freeman GJ (2008). PD-1 and its ligands in tolerance and immunity. Annu Rev Immunol.

[24] Sznol M, Chen L (2013). Antagonist antibodies to PD-1 and B7–H1 (PD-L1) in the treatment of advanced human cancer. Clin Cancer Res.

[25] Facchinetti F, Marabelle A, Rossi G (2016). Moving immune checkpoint blockade in thoracic tumors beyond NSCLC. J Thorac Oncol.

[26] Zhang B, Liu Y, Zhou S (2020). Predictive effect of PD-L1 expression for immune checkpoint inhibitor (PD-1/PD-L1 inhibitors) treatment for non-small cell lung cancer: a meta-analysis. Int Immunopharmacol.

[27] Liu Y, Zhou S, Du Y (2019). Efficacy and safety of programmed death 1 inhibitors in patients with advanced non-small cell lung cancer: a meta-analysis. Cancer Manag Res.

[28] Schreiber RD, Old LJ, Smyth MJ (2011). Cancer immunoediting: integrating immunity’s roles in cancer suppression and promotion. Science.

[29] Larkin JMG, Chiarion-Sileni V, Gonzalez R (2019). 5-year survival outcomes of the CheckMate 067 phase III trial of nivolumab plus ipilimumab (NIVO+IPI) combination therapy in advanced melanoma. Ann Oncol.

[30] Menshawy A, Eltonob AA, Barkat SA (2018). Nivolumab monotherapy or in combination with ipilimumab for metastatic melanoma: systematic review and meta-analysis of randomized-controlled trials. Melanoma Res.

[31] Rizvi NA, Cho BC, Reinmuth N (2020). Durvalumab with or without tremelimumab vs standard chemotherapy in first-line treatment of metastatic non-small cell lung cancer: the MYSTIC phase 3 randomized clinical trial. JAMA Oncol.

[32] Planchard D, Reinmuth N, Orlov S (2020). ARCTIC: durvalumab with or without tremelimumab as third-line or later treatment of metastatic non-small-cell lung cancer. Ann Oncol.

[33] Ready N, Hellmann MD, Awad MM (2019). First-line nivolumab plus ipilimumab in advanced non-small-cell lung cancer (CheckMate 568): outcomes by programmed death ligand 1 and tumor mutational burden as biomarkers. J Clin Oncol.

[34] Borghaei H, Paz-Ares L, Horn L (2015). Nivolumab versus docetaxel in advanced nonsquamous non-small-cell lung cancer. N Engl J Med.

[35] Brahmer J, Reckamp KL, Baas P (2015). Nivolumab versus docetaxel in advanced squamous-cell non-small-cell lung cancer. N Engl J Med.

[36] Carbone DP, Reck M, Paz-Ares L (2017). First-line nivolumab in stage IV or recurrent non-small-cell lung cancer. N Engl J Med.

[37] Hellmann MD, Ciuleanu TE, Pluzanski A (2018). Nivolumab plus ipilimumab in lung cancer with a high tumor mutational burden. N Engl J Med.

[38] Wu YL, Lu S, Cheng Y (2019). Nivolumab Versus docetaxel in a predominantly chinese patient population with previously treated advanced NSCLC: CheckMate 078 randomized phase III clinical trial. J Thorac Oncol.

[39] Hellmann MD, Rizvi NA, Goldman JW (2017). Nivolumab plus ipilimumab as first-line treatment for advanced non-small-cell lung cancer (CheckMate 012): results of an open-label, phase 1, multicohort study. Lancet Oncol.

[40] Bazhenova L, Redman MW, Gettinger SN (2019). A phase III randomized study of nivolumab plus ipilimumab versus nivolumab for previously treated patients with stage IV squamous cell lung cancer and no matching biomarker (Lung-MAP Sub-Study S1400I, NCT02785952). J Clin Oncol.

