# Correction to: Nivolumab monotherapy or combination therapy with ipilimumab for lung cancer: a systemic review and meta-analysis

**DOI:** 10.1186/s12935-021-02220-3

**Published:** 2021-10-04

**Authors:** Huihui Jiang, Aiqun Xu, Wanli Xia, Xingyuan Xia, Pulin Li, Binbin Zhang, Ke Zhu, Sijing Zhou, Ran Wang

**Affiliations:** 1 Department of Respiratory and Critical Care Medicine, The First Affiliated Hospital of Anhui Medical University, Hefei, 230022 China; 2 Department of General Medicine, Hefei Second People’s Hospital, Hefei, 230001 China; 3 Department of Thoracic Surgery, The First Affiliated Hospital of Anhui Medical University, Hefei, 230022 China; 4 Hefei Third Clinical College of Anhui Medical University, Hefei, 230022 China; 5 Hefei Prevention and Treatment Center for Occupational Diseases, Hefei, 230022 China

## Correction to: Cancer Cell Int (2021) 21:426 https://doi.org/10.1186/s12935-021-02100-w

Following the publication of the original article [1], we were notified that the affiliation of the third author was incorrect.

- Originally published affiliation: Department of Oncology, The First Affiliated Hospital of Anhui Medical University, Hefei 230022, China
- Corrected affiliation: Department of Thoracic Surgery, The First Affiliated Hospital of Anhui Medical University, Hefei 230022, China

The original article has been corrected.
